# National and Local Influenza Surveillance through Twitter: An Analysis of the 2012-2013 Influenza Epidemic

**DOI:** 10.1371/journal.pone.0083672

**Published:** 2013-12-09

**Authors:** David A. Broniatowski, Michael J. Paul, Mark Dredze

**Affiliations:** 1 Department of Engineering Management and Systems Engineering, The George Washington University, Washington, District of Columbia, United States of America; 2 Center for Advanced Modeling in the Social, Behavioral, and Health Sciences, Department of Emergency Medicine, School of Medicine, Johns Hopkins University, Baltimore, Maryland, United States of America; 3 Department of Computer Science and Center for Language and Speech Processing, Johns Hopkins University, Baltimore, Maryland, United States of America; 4 Human Language Technology Center of Excellence and Department of Computer Science, Johns Hopkins University, Baltimore, Maryland, United States of America; University of Warwick, United Kingdom

## Abstract

Social media have been proposed as a data source for influenza surveillance because they have the potential to offer real-time access to millions of short, geographically localized messages containing information regarding personal well-being. However, accuracy of social media surveillance systems declines with media attention because media attention increases “chatter” – messages that are about influenza but that do not pertain to an actual infection – masking signs of true influenza prevalence. This paper summarizes our recently developed influenza infection detection algorithm that automatically distinguishes relevant tweets from other chatter, and we describe our current influenza surveillance system which was actively deployed during the full 2012-2013 influenza season. Our objective was to analyze the performance of this system during the most recent 2012–2013 influenza season and to analyze the performance at multiple levels of geographic granularity, unlike past studies that focused on national or regional surveillance. Our system’s influenza prevalence estimates were strongly correlated with surveillance data from the Centers for Disease Control and Prevention for the United States (r = 0.93, p < 0.001) as well as surveillance data from the Department of Health and Mental Hygiene of New York City (r = 0.88, p < 0.001). Our system detected the weekly change in direction (increasing or decreasing) of influenza prevalence with 85% accuracy, a nearly twofold increase over a simpler model, demonstrating the utility of explicitly distinguishing infection tweets from other chatter.

## Introduction

Health organizations require accurate and timely disease surveillance techniques in order respond to emerging epidemics. Such information may inform planning for surges in patient visits, therapeutic supplies, and public health information dissemination campaigns [1]. Nevertheless, collecting and aggregating the information required to accurately report the spread of any disease is time and labor intensive. For example, the U.S. Centers for Disease Control and Prevention (CDC) collects and aggregates data from one of the most effective disease surveillance systems: a network of 2,700 outpatient providers across the United States that provides counts of influenza-like illness (ILI) rates, and weekly reports are issued summarizing data from each previous week [2]. 

There has been increasing interest in using social media and other Internet resources to perform disease surveillance. For example, news articles, search engine statistics and mobile phone data have been shown to be informative indicators of influenza activity, including the novel H7N9 outbreak [3,4]. In contrast to contemporary data collection methods, social media have the potential to allow public health officials to respond to disease outbreaks in real time. Social media is often tagged by geographic location (geo-located) potentially providing actionable information to policymakers at municipal, as well as national, health agencies. To the extent that these systems are accurate, they have the potential to revolutionize disease surveillance. Although social media disease surveillance systems have shown significant promise, to date, their potential has not been realized. 

Much of the literature on disease surveillance using social media has focused on tracking influenza with Twitter. Twitter is a popular social media website, especially appealing as a data source because it offers nearly instant access to millions of public short status message per day, known as “tweets.” These messages often contain information regarding personal well-being. A number of academic groups and startup companies have attempted to leverage social media illness reports to generate forecasts and estimates of disease prevalence. For example, the senior author has previously demonstrated that tweets can be correlated with publicly available influenza data from the CDC [5].

Whereas several researchers have correlated social media signals with influenza prevalence metrics in a retrospective fashion (e.g., 6-9), in this paper we demonstrate influenza surveillance using social media with a system built and deployed before the influenza season started. In addition, our approach is the first to have been tested successfully at both the national and municipal levels. We are the first to have both reported comprehensive results from this past year’s most recent influenza epidemic and to have a blind system evaluation conducted by a municipal health agency. 

We have found that the accuracy of most social media surveillance systems declines with media attention. This is because media attention increases Twitter “chatter” – tweets that are about the flu but that do not pertain to an actual infection. These messages can mask signs of actual infection. Examples include tweets indicating awareness of flu (e.g. “I hope I don’t get the flu”) and reports of a celebrity’s flu. Disproportionate media attention has similarly resulted in prevalence overestimates in Google Flu Trends, another web-based flu surveillance system [10]. Commonly used Twitter techniques, such as keyword matching or linear regression – which were shown to correlate with CDC data during the 2009 pandemic – have not been strongly correlated with infection data in more recent seasons [9]. The example above illustrates how tweets can be misleading: the message is clearly about the flu but is not about an infection. Previously used techniques do not explicitly attempt to differentiate between chatter and actual infection. The technique presented in this paper successfully filters out tweets that are not reporting influenza infection.

## Methods

**Figure 1:**
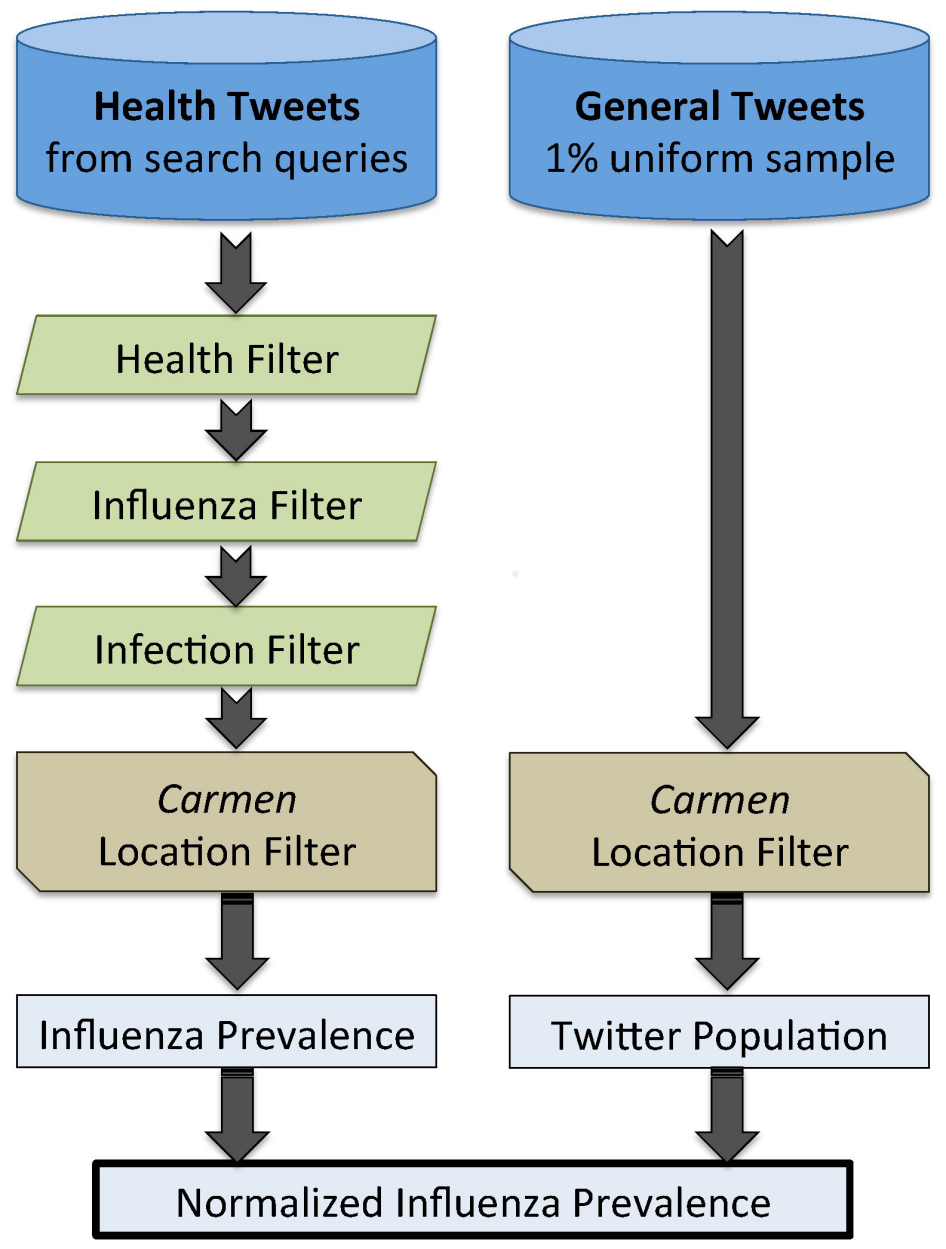
System for estimating influenza prevalence from Twitter. This figure shows the components of our system for estimating influenza prevalence from Twitter. A stream of tweets matching hundreds of health-related keywords is passed through three classification filters to remove irrelevant tweets. The locations of tweets are identified with our geolocation system, Carmen, and only tweets in the location of interest are saved. The volume of tweets is normalized by the total volume of tweets from a random sample of Twitter to produce a prevalence measure.

We have created a new supervised classification model that overcomes this barrier by separating tweets indicating influenza infection from those that indicate influenza awareness or concern. This model, along with additional classifiers and a new geolocation system, are used to estimate influenza prevalence from normalized tweet volume. The various components of our system are summarized by the diagram in Figure 1.

### Data Collection

Twitter provides tools to access ongoing “streams” of public data that can be downloaded at no cost. This study uses data downloaded from two such streams. The first stream, which we call the “general” stream, represents a one percent uniform sample of all Twitter messages. The second is a custom stream, called the “health” stream, which downloads only tweets containing any of 269 health-related keywords that we provided. The health stream is also limited to one percent of total Twitter volume, but this cap is applied to the subset of tweets matching the health keywords, allowing us to collect a much larger amount of relevant data from this source. The sampling is uniformly applied to this entire subset of health tweets, so relative rises in influenza-related tweets will be represented in the sample, even though the data size is fixed at the one percent limit.

Our data set for this study begins September 30, 2012, the start of the first week of the 2012-2013 influenza season as defined by the CDC [11], through May 31, 2013. This collection contains 1.0 billion tweets from the general stream and 0.3 billion tweets identified as health related, using a classifier described in the next section, from the health stream, which is of similar volume as the general stream.

### Data Filtering

We used a staged-approach to data filtering. We used binary classification models to identify relevant data for influenza surveillance at each stage. These models indicated whether tweets were relevant to health, relevant to influenza, and indicative of an actual infection. The first filter – distinguishing tweets that are relevant or irrelevant to health – utilizes a combination of keyword filtering and a support vector machine (SVM) trained on 5,128 tweets that were annotated with these two categories. This classifier was estimated to have 90% precision and 32% recall through 10-fold cross-validation. A bias towards precision is justified since we can still obtain large samples of data. The full details are described in an earlier technical report [12]. The two influenza filters were developed recently for the specific purpose of influenza surveillance. 11,990 tweets were labeled with three types of influenza-related codes: (1) whether the tweet discussed influenza or not, (2) whether an influenza tweet indicated an infection or is merely an acknowledgement of the user's awareness of influenza, and (3) whether an influenza infection tweet was in reference to the author of the tweet or another person (this third type of information was not used in our final classifiers). The labeled data were used to train parameters of separate logistic regression models for the two classification tasks. The features (covariates) of the model included the words in the tweet, all sequential phrases composed of two to three words (n-grams), and other linguistic information regarding the message semantics, syntax, and writing style. These linguistic features are described in detail in our recent paper [9]. The human-annotated data used to train these models came from the 2009–2010 and 2011–2012 influenza seasons. The two classifiers were respectively estimated to have 67% and 74% precision and 87% and 87% recall through 10-fold cross-validation, using the classification thresholds with maximal F1 score, favoring a combination of both precision and recall.

Using this pipeline classification system, we identified 570,000 influenza infection tweets during the eight months of data collection. We then normalized the weekly number of such infection tweets by the total number of tweets in the general stream for that week to produce a Twitter-based influenza prevalence measure. To evaluate our prevalence estimate, we compared the resulting measure to the CDC’s US Outpatient Influenza-Like Illness Surveillance Network, which includes the number of visits for influenza-like illness (ILI). 

### Location Filtering

An important aspect of accurate social media surveillance is identifying the geographic location of each tweet [13]. To estimate the influenza prevalence in a geographic region, we only included tweets from that region. We used our recently developed geolocation system, called Carmen, to identify the location of 22% of the tweets in our collection [14]. In addition to the GPS information associated with a small percentage of the tweets, Carmen utilizes information from the users’ public biographic profiles. Over half of the user biographies include a self-reported location, which can contain values such as “New York, NY” and “NYC,” as well as nonsensical values such as “Candy Land.” Our system resolves these values to a country, state, county, and city, while resolving aliases of the same place and filtering out nonsense locations. In an evaluation set of 56,000 tweets, the two locations considered in our experiments – the United States and New York City – were resolved with respective accuracies of 92% and 61% (to within 50 miles of NYC).

### Data Exclusion

Due to system outages, we had incomplete data collection during the days of 10/11/12—10/17/12. The outages affected the health stream but not the general stream, which would lead to an incorrect calculation of the prevalence on those days. We therefore excluded those days from the calculation of the weekly rates for the weeks beginning 10/7/12 and 10/14/12. The rate for the week of 5/26/13 excludes the day of 6/01/13 because that is last day of data collected before beginning this study.

## Results

### National Level: United States

**Figure 2:**
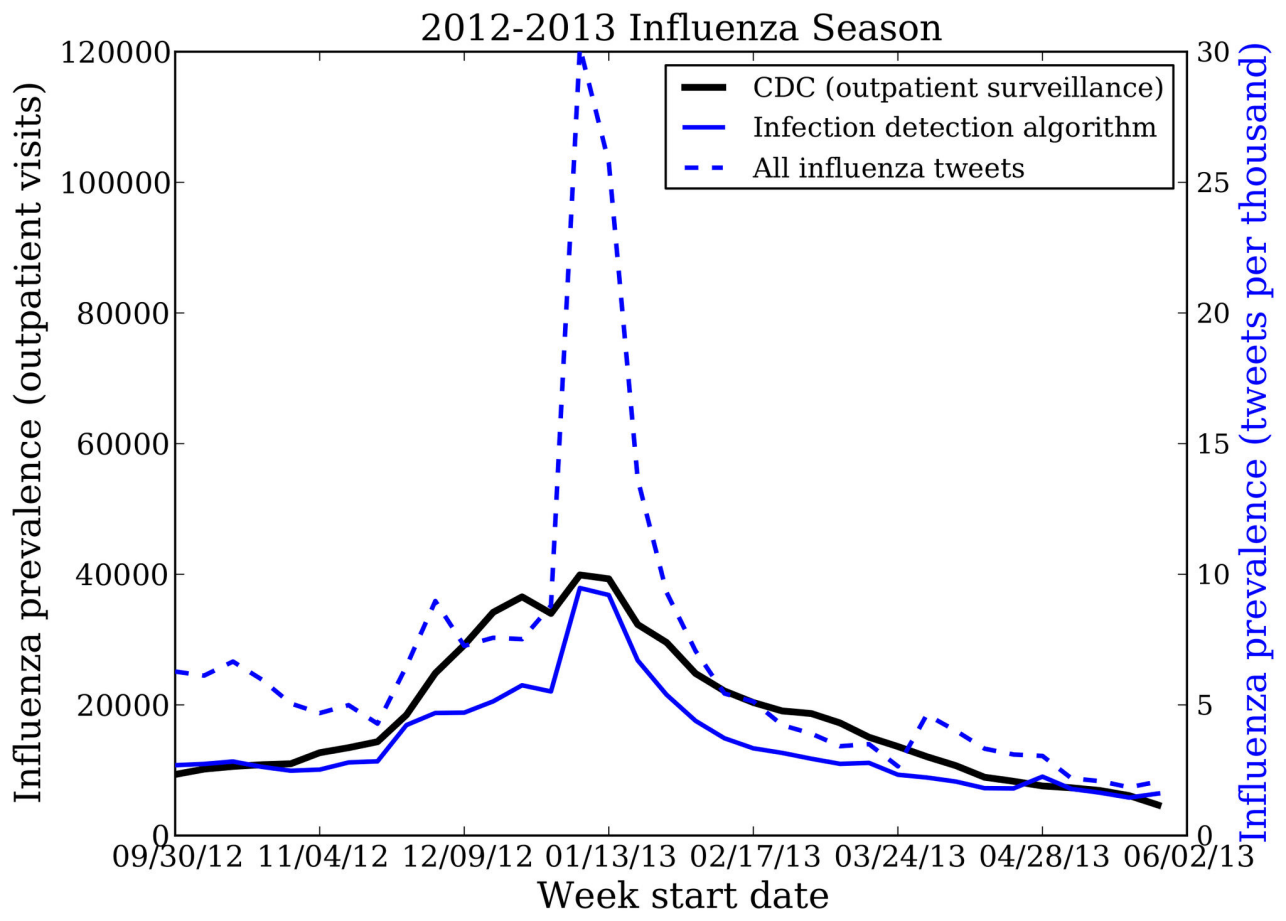
2012-2013 national influenza rates from Twitter and CDC surveillance. This figure shows national influenza rates of the United States as predicted by two Twitter-based algorithms alongside the influenza-like illness surveillance network data from the US Centers for Disease Control and Prevention (CDC). The dashed blue line is the measure estimated by a simple model of keyword matching, while the solid blue line is the measure estimated by our new infection detection model. Our new algorithm more closely matches the CDC data (solid black line), while the simpler keyword model infers spurious spikes due to other Twitter chatter, e.g. in early December and early April.

Our system identified 104,200 influenza infection tweets from the United States. The weekly number of tweets indicating influenza infection is strongly correlated with weekly CDC ILI outpatient counts from October 2012 - May 2013 (r = 0.93; p < 0.001). In contrast, the weekly number of tweets containing influenza keywords provided by the US Department of Health and Human Services is much less strongly correlated with patient illness data (r = 0.75; p < 0.001). The difference between these correlations is significant at the p<0.001 level. The mean absolute error of the keyword-based estimates is 0.0102 after normalizing the weekly rates to sum to 1. The mean absolute error of our infection estimates is 0.0046, a 45% reduction in error over the keyword filter. The national estimates produced by the two Twitter algorithms are shown alongside the CDC rates in Figure 2.

### Municipal Level: New York City

The same technique also successfully captures influenza trends at the level of a municipality, demonstrated on 4,800 influenza infection tweets identified from New York City. The New York City Department of Health and Mental Hygiene carried out a blind evaluation of our algorithm, yielding a strong correlation between the city’s weekly emergency department visits for ILI and the number of New York City weekly tweets indicating influenza infection (r = 0.88; p < 0.001). The simpler keyword-based system was less strongly correlated (r = 0.72; p < 0.001). This evaluation was conducted on data spanning the same period of time as the national evaluation.

### Real-Time Response

We performed weekly correlations, from the beginning of December through the end of February, between the national ILI data beginning in the first week of October – the start of the influenza season – with Twitter infection surveillance data. The Pearson correlation coefficient ranged from 0.91 to 0.97, with a mean of 0.93 and standard deviation of 0.02. Additionally, in the thirteen weeks in which the national influenza rate had a larger-than-average change from the previous week, our system matched the direction of the change (increasing or decreasing) with 85% accuracy, in contrast to 46% accuracy by the baseline keyword-based system.

### Time Series Analysis: Differencing and Cross-Correlation

Previous studies on this topic have focused on the types of correlations described above. In this regard our system performs at or above the level of any previous system. In this section, we go beyond previous work and include an in depth statistical analysis of the characteristics of the data.

Any correlation analysis of time series data is potentially subject to bias if the underlying data are not stationary – i.e., if their distributional properties change over time. For example, if each week’s influenza infection count is a function of the previous week’s count, then we would expect a correlation between any two measures that capture the same overall trend. There is no reason to believe, *a priori*, that Twitter data should be correlated with actual influenza infection, therefore success at matching the overall shape of the influenza trend would be a success of our method. Nevertheless, one might argue that any curve that increases to some peak and then decreases would have some degree of correlation with CDC data. To test this hypothesis, we perform a more in-depth time series analysis in order to examine whether we can also detect deviations from this larger seasonal trend.

An examination of the autocorrelation functions for the national CDC, Twitter infection, and Twitter keywords data sources indicated a gradual reduction with lag, consistent with a nonstationary process. Our subsequent analysis therefore followed the Box-Jenkins procedure [15]. We examined the partial autocorrelation function and found significant effects at the p<0.05 level for a lag of one week. Lags of two weeks or greater were not significant at the p<0.05 level. Therefore, first-order differencing was applied to each of these data sets – i.e., we examined the difference between each week's and the previous week's numbers. An examination of the partial autocorrelation function for the first-order-differenced CDC data still yielded a correlation at a lag of 1 week that was significant at the p<0.05 level. Therefore, second order differencing was applied to each data set – i.e., the same differencing technique was applied to the first-order-differenced data. Examination of the associated autocorrelation and partial autocorrelation functions indicated that all lags greater than zero were not significant at the p=0.05 level, consistent with a stationary process. 

We next examined the correlation between the second-order-differenced versions of the CDC, Twitter infection, and Twitter keywords data. The infection classifier data were more strongly correlated (r=0.82; p<0.001) with the CDC data than were the keywords classifier data (r=0.78; p<0.001), although this difference is not significant at the p=0.05 level. Normal probability plots of the associated residuals for each of these two correlations were found to be indicative of normality. In addition, the autocorrelation functions of both sets of residuals were examined, and lags greater than zero were found not to be significant at the p=0.05 level.

**Figure 3:**
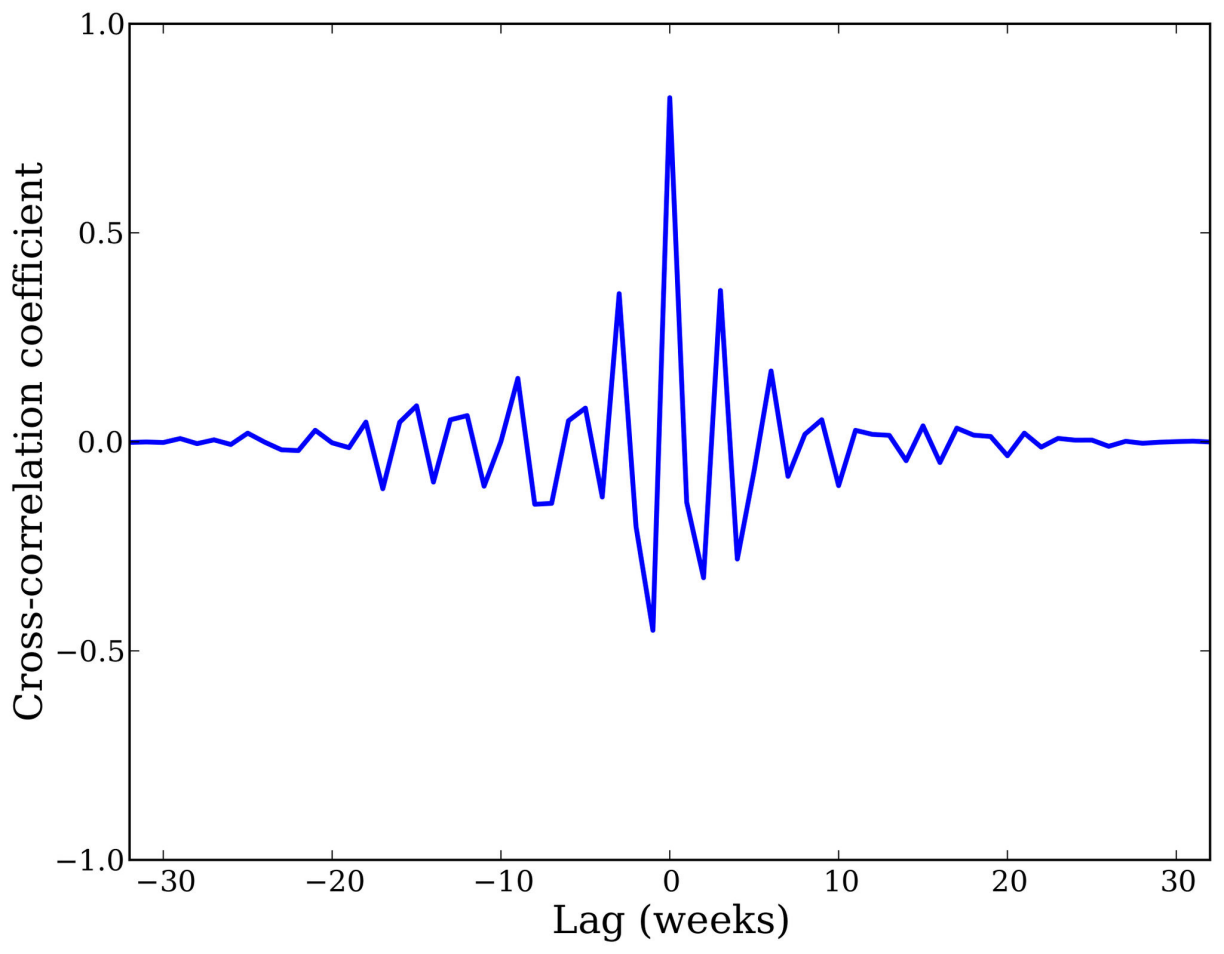
Cross-correlation between Twitter infection rates and CDC ILI rates. This figure shows the cross-correlation function for the residuals of the second-order-differenced CDC and Twitter infection data, as described in the “Time Series Analysis” section. These results show that the Twitter estimates neither lead nor lag the CDC ILI rates, although the Twitter data are publicly available up to two weeks earlier than CDC data.

Finally, we examined the cross-correlation functions for the original and differenced data sources. Cross-correlation results for the non-differenced data sources were dominated by the seasonal influenza trend and were therefore not informative beyond indicating a peak is at zero, indicating that the Twitter data neither lags nor leads the CDC data. A similar evaluation with the New York City data found that the Twitter data neither lags nor leads the city’s ILI data. The cross-correlation function for the residuals of the second-order-differenced national CDC and infection data is shown in Figure 3. 

This additional time series analysis shows that even if we were to put in a curve that peaked during the peak of flu season (i.e., had no lag), and whose derivative matched the slope of the CDC data, we would still be contributing useful information by showing how deviations from this overall trend occur. In other words, the additional analysis shows that we can capture the detail beyond the overall trend; however, we often do not know when flu season will peak in general and the seasonality in influenza infection is not so well understood [16]. For example, the 2011-2012 flu season was quite mild and lacked a strong peak, whereas the 2009 H1N1 epidemic had unusual patterns outside the seasonal norm. Our method has therefore demonstrated the ability to match both the seasonal trend as well as residual deviations from that trend.

## Discussion

Our new algorithm demonstrates significant improvements and is less sensitive to Twitter chatter by focusing on reports of actual influenza infection. For example, on December 3, 2012, the CDC issued a press release [17] concerning influenza, leading to a spike in the number of influenza tweets (see Figure 2) that was not correlated with actual influenza infection. Similarly, around January 1, many news outlets ran flu epidemic stories. 

Finally, on April 5, the CDC held a press conference [18] about the H7N9 “bird flu” virus in China, which garnered significant media attention. In each of these cases, we observed a large increase in the number of tweets with influenza keywords (dashed blue line) concurrent with the media coverage. In contrast, our “infection only” system (solid blue line) increases only slightly or not at all, demonstrating our ability to ignore increases due to news coverage and other sources of chatter. 

Our ability to filter tweets indicating influenza infection from other tweets at the level of a municipality represents an additional novel technical advance by providing accurate localized Twitter-based influenza tracking. 

As this influenza season progressed, the Twitter infection surveillance system consistently predicted the CDC data through the most recent week. Our Twitter system strongly correlates with government data throughout all weeks of the season, and can thus be used for real-time analysis in addition to retrospective analysis.

There are inherent limitations in using social media to make inferences about the real world. One of these is coverage. While we were able to produce reliable metrics for New York City, smaller municipalities may not have enough Twitter users to produce robust prevalence estimates. Additionally, much of the world is currently excluded from the current version our system, which can only process English-language tweets. However, these limitations do not prohibit the creation of similar systems for other languages. One may question the representativeness of our approach since the groups most susceptible to influenza infection – the elderly and young children – tend to be underrepresented on Twitter; however, for the purpose of identifying general population-level trends, this sample bias may not have a large effect. Furthermore, our experimental results demonstrated that we can accurately infer trends despite these drawbacks. We expect these types of limitations to diminish over time as social media becomes more widely adopted.

## Conclusion

Using Twitter data from October 2012 - May 2013, we have been able to differentiate between reports of actual infection and Twitter chatter. Our infection curve correlates strongly with CDC ILI data. In addition, we have demonstrated the ability to use the same technique on the municipal level by correlating with ILI data in New York City. Our infection detection algorithm is consistently able to predict the direction of CDC data in weekly increments. Finally, the in-depth statistical analysis of our results demonstrates that our estimates are significant over general seasonal trends. These findings are significant advances over available algorithms in terms of accuracy, level of geographic granularity, and decision-based evaluations.

We anticipate that our technique will inform clinical practice and health policy, especially as regards influenza surveillance techniques. Real-time tools such as our system have the potential to enable clinicians to anticipate the need for surges in influenza-like illness up to two weeks in advance of existing data collection strategies [19]. Early knowledge of an upward trend in disease prevalence can inform patient capacity preparations [1] and increased efforts to distribute the appropriate vaccine or other treatment, whereas knowledge of a downward trend can signal the effectiveness of these efforts. In addition, policymakers may use such data sources to track the spread of influenza at national and municipal levels. Our analysis demonstrates that these techniques can be applied adaptively in real time.
